# Automatic Ability Attribution after Failure: A Dual Process View of Achievement Attribution

**DOI:** 10.1371/journal.pone.0063066

**Published:** 2013-05-07

**Authors:** Michiko Sakaki, Kou Murayama

**Affiliations:** 1 Davis School of Gerontology, University of Southern California, Los Angeles, California, United States of America; 2 Department of Psychology, University of California Los Angeles, Los Angeles, California, United States of America; University of Bologna, Italy

## Abstract

Causal attribution has been one of the most influential frameworks in the literature of achievement motivation, but previous studies considered achievement attribution as relatively deliberate and effortful processes. In the current study, we tested the hypothesis that people automatically attribute their achievement failure to their ability, but reduce the ability attribution in a controlled manner. To address this hypothesis, we measured participants’ causal attribution belief for their task failure either under the cognitive load (load condition) or with full attention (no-load condition). Across two studies, participants attributed task performance to their ability more in the load than in the no-load condition. The increased ability attribution under cognitive load further affected intrinsic motivation. These results indicate that cognitive resources available after feedback play crucial roles in determining causal attribution belief, as well as achievement motivations.

## Introduction

Causal attribution has been one of the most influential frameworks for understanding how individuals perceive and interpret their own behaviors as well as other’s behaviors. In the realm of achievement motivation, since Weiner and Kukla’s influential work [1], a vast amount of research has addressed motivational and emotional consequences of perceived causes (e.g., ability, task difficulty) in achievement contexts [2]–[4]. Researchers have also revealed various factors influencing causal attribution of one’s achievement performance (for reviews see [5]–[6]). Despite the extensive previous research, however, past studies have been relatively mute on the underlying process of causal attribution in achievement settings. In the current study, drawing on the dual process models of social cognition [7], we examine how people’s causal attribution of their performance is associated with automatic- and controlled- processes.

Ability is one of the primary concerns of people in achievement settings [8]. People are motivated to seek ability information [9] and are chronically concerned with how their ability is evaluated [10]. One’s belief about ability also alters achievement behaviors [11]–[12] and reactions to performance feedback [13]. Furthermore, threats on people’s ability impair performance in a wide variety of tasks [14]. Because of the strong saliency of ability, it seems to be the most straightforward factor in which one attributes his/her failure when faced with achievement feedback [15].

However, attributing task failure to one’s ability can lead to maladaptive outcomes. Ability is an internal and stable causal factor that people cannot control; thus ability attribution following task failure tends to augment one’s expectation for future failures, demotivating people from the task [4]. Indeed, ability attributions for failure are known to lower motivation and self-esteem, and increase depressive symptoms as well as negative affects [3], [16]. Importantly, past studies indicated that people are aware of those maladaptive consequences of ability attribution [17]–[18]. This suggests the possibility that people have an inclination to reduce ability attributions when facing failure outcomes.

In the current study, we hypothesized that people automatically attribute their task performance to their ability, but reduce ability attribution in a controlled manner. We addressed separate contributions of automatic and controlled processes by manipulating participants’ cognitive resources [19]. During the study, we measured participants’ causal attribution of their failure while manipulating their cognitive resources; Half of participants were told to infer causal reasons under cognitive load (load condition), whereas the other made an attribution with full attention (no-load condition). If ability attribution occurs automatically and is reduced in a controlled manner, ability attribution should be enhanced in the load than in the no-load condition. Study 1 provided the evidence for this hypothesis. Study 2 replicated the automatic ability attribution even when ability was not a plausible cause for failure. Study 2 also documented motivational consequences of ability attribution by assessing participants’ motivation about achievement tasks.

## Study 1

### Method

Thirty-three Japanese undergraduates (*M*_age_ = 20.38; 15 males) were randomly assigned to the load or no-load condition. The experimental protocol (both Studies 1 and 2) conformed to the ethics guidelines of the Japanese Psychological Association. The study was approved by the ethics committee of the Graduate School of Education at the University of Tokyo and participants provided their written consent. All data were analyzed anonymously; data files are available on request.

Participants were given a modified version of the Raven’s progressive matrices task for 10 min. The task consisted of 10 difficult items; no one could solve all of the items. Immediately after the task, a computer provided bogus success feedback: “Your score is 28 (the average score from undergrads in your university is 54).” All participants then answered a manipulation check question (“How well did you perform on the reasoning test?”) on a 4-point scale (1: failed, 2: relatively failed, 3: relatively succeeded, 4: succeeded). Both in Studies 1 and 2, most participants reported in the manipulation check question that they failed or relatively failed at the task (Study 1: *M* = 1.67, SD = 0.69; Study 2: *M* = 1.67, SD = 0.48), but two participants in Study 1 and four participants in Study 2 reported that they succeeded or relatively succeeded on the task. Because the causal reasoning questionnaire was designed only for failure situations, we did not conduct the causal reasoning questionnaire to these participants; therefore, they were not included in the reported analyses.

Participants were then given a causal reasoning questionnaire [20]–[21] and rated the extent to which each item caused their failure on a 5-point scale. The questionnaire included four items on ability attribution (e.g., “because of the lack of my reasoning ability,” “because I’m not smart”; reliability = .87) and four items on task attribution (e.g., “because the task was difficult,” “because the test was too advanced”; reliability = .84).

Participants in the no-load condition answered the attribution questionnaire without any additional tasks. In contrast, participants in the load condition answered the questionnaires while working on a sound-pitch judgment task obtained from previous research [19]. In the sound judgment task, participants listened to a sound sequence which consisted of a low-, a medium-, and a high- pitched tone in randomized order with variable intervals (approx 1–3 secs). The participants’ task was to keep track of the sound sequence and press a button immediately after detecting a sequence of low-, medium-, and high- pitched tones in that order. Participants were familiarized with this dual task procedure using a different questionnaire at the beginning of the study.

### Results

**Figure 1:**
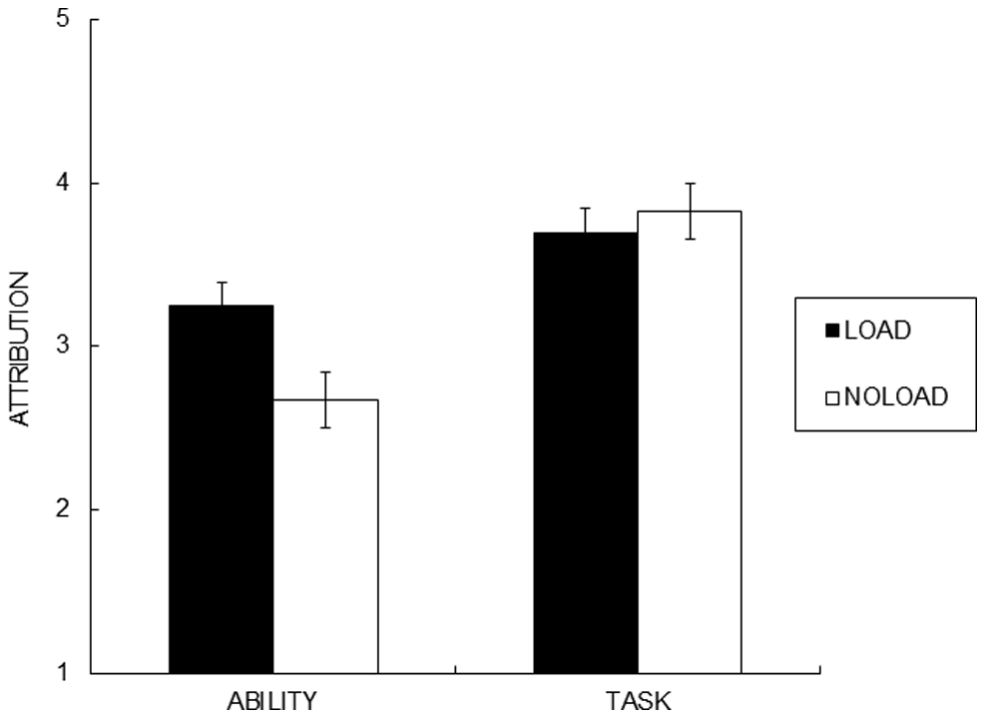
Ability and task attribution made under full and divided attention condition after participants received failure feedback (Study 1). Ability attribution score was higher in the load condition than in the no-load condition, while task attribution score did not differ across attention manipulation. Error bars represent standard errors.

Generalized Eta Squared (*η^2^_G_* ) was used to estimate effect sizes [22]. One participant was identified as an outlier and excluded from the analysis (i.e., Tukey’s criterion of three times the interquartile range higher than the third quartile. Grubbs’ test for outliers was significant [23]). As shown in Figure 1, task load manipulation selectively enhanced ability attribution. A 2 (cause: task, ability) X 2 (attention) mixed ANOVA with cause as a repeated measures factor on the attribution scores revealed a significant cause x attention interaction, *F*(1, 28) = 4.86, *η^2^_ G_ = *.05, *p*<.05. Participants reported higher ability attribution in the load than in the no-load condition (Figure 1), *F*(1, 28) = 4.65, *p*<.05, while the task attribution score did not differ across the conditions (*p*>.78).

## Study 2

Study 2 sought to extend Study 1 in two ways. First, we examined whether our findings can be replicated even when ability was not a plausible cause for failure. Specifically, participants were explicitly instructed that the task was unusually difficult when they are provided failure feedback. Thus, participants did not have any plausible reasons to believe that the failure was caused by their lack of ability, which should allow for a stronger test for the automaticity of ability attribution. Second, Study 2 assessed intrinsic motivation after the task to address motivational consequences of ability attribution. Given that ability attribution after failure is associated with maladaptive outcomes, we could expect that increased ability attribution under cognitive load should reduce intrinsic motivations toward a task. In other words, we hypothesized that ability attribution serves as a mediator between cognitive load manipulation and intrinsic motivation.

### Method

Twenty-eight Japanese undergraduates (*M*_age_ = 19.8; 20 males) were randomly assigned to the load or no-load condition. The procedure was identical to Study 1, with two exceptions. First, the feedback included only participants’ raw score without the average score of other students (i.e., “Your score is 28”), and participants were explicitly told that the task was designed to be extremely difficult for all students. Second, along with the causal attribution measurement (reliability: ability = .97, task = .86), participants answered five items on intrinsic motivation to the reasoning task with a 5-point scale (reliability = .92; e.g., “I found the task interesting” [24]).

### Results

**Figure 2:**
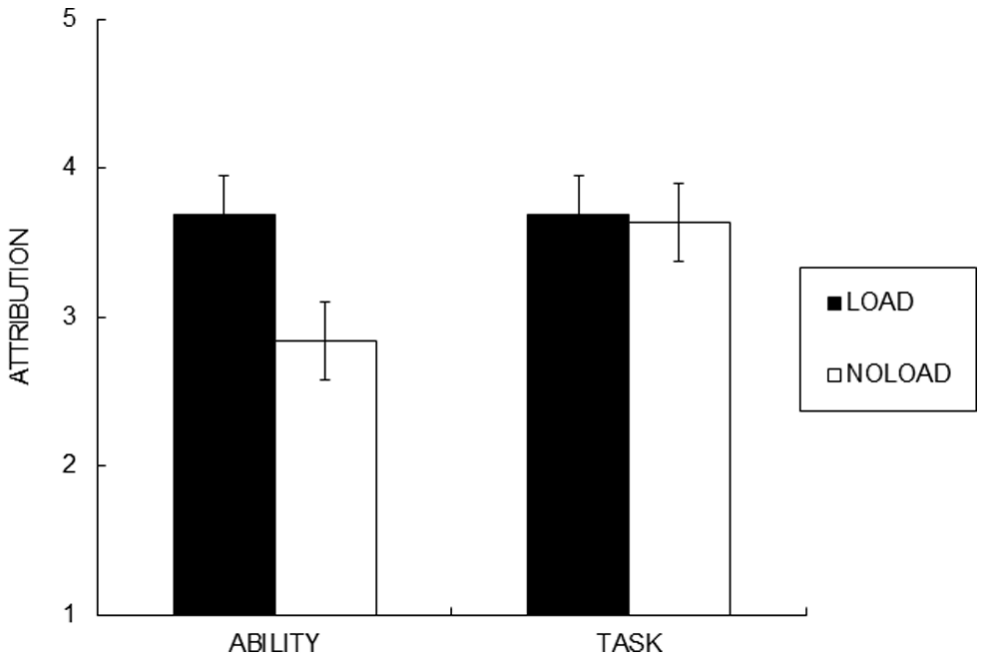
Ability and task attribution made under full and divided attention condition after participants received failure feedback (Study 2). Ability attribution score was higher in the load condition than in the no-load condition, even when ability is not a plausible cause for failure. Task attribution score did not differ across attention manipulation. Error bars represent standard errors.

As indicated in Figure 2, task load manipulation again selectively enhanced ability attribution. A 2 (cause) X 2 (attention) ANOVA with cause as a repeated measures factor on the attribution scores revealed a main effect of cause, *F*(1, 22) = 4.85, *η^2^_Gs_* = .07, *p*<.05, and a significant cause and attention interaction (Figure 2), *F*(1, 22) = 4.85, *η^2^_Gs_* = .07, *p*<.05. Consistent with Study 1, participants reported higher ability attribution in the load than in the no-load condition, *F*(1, 22) = 8.09, *p*<.01, but the task attribution score did not differ across the conditions (*p*>.80).

**Figure 3:**
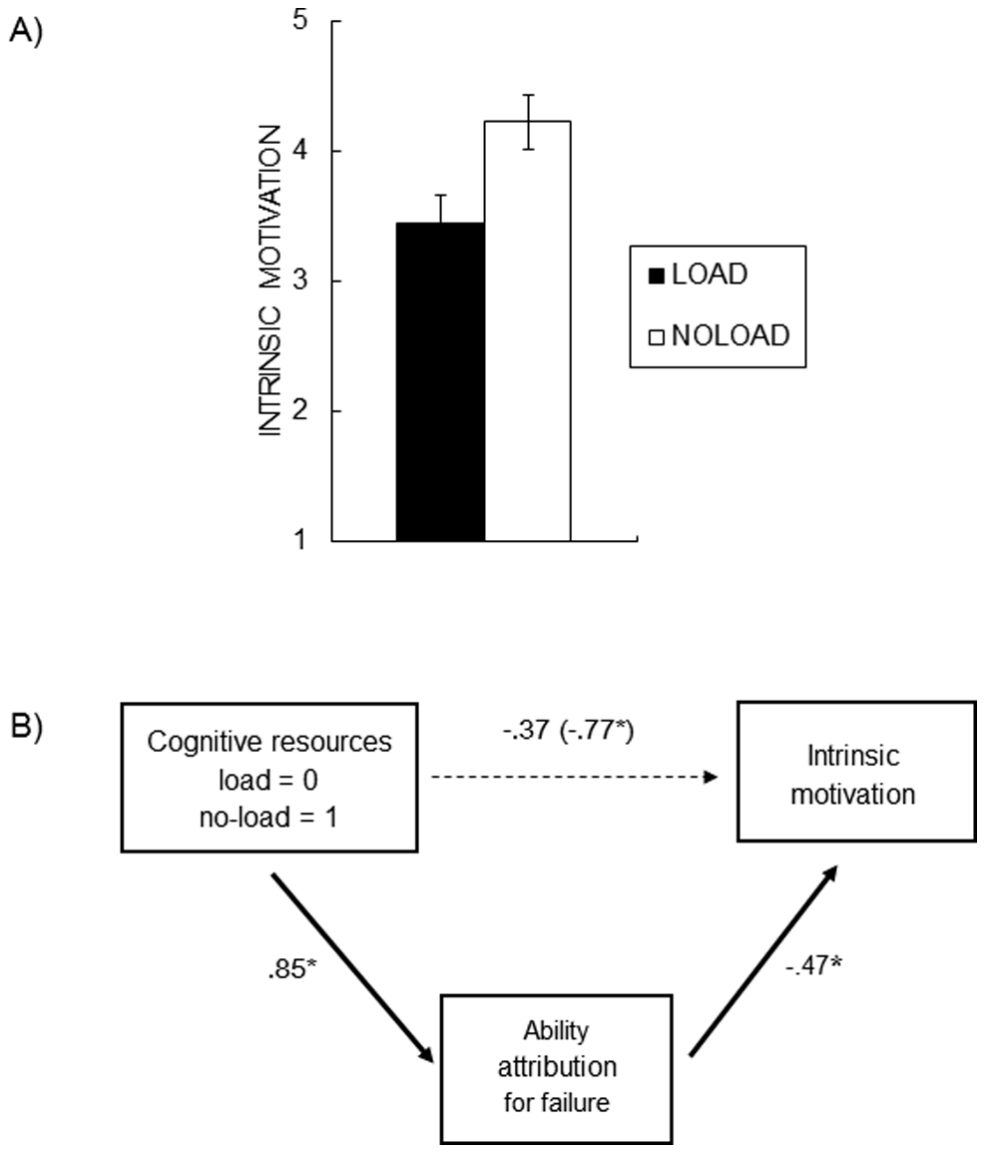
(A) The intrinsic motivation about the reasoning task was decreased in the load condition than in the no-load condition after failure. (B) The effects of cognitive resources on the motivation were fully mediated by the ability attribution. Solid lines represent significant paths and a dashed line represents a non-significant effect.

Additional analysis (Figure 3A) showed that participants showed decreased intrinsic motivation in the load than in the no-load condition, *F*(1, 22) = 6.04, *η^2^_G_ = *.22, *p*<.05. To address whether the reduced motivation in the load condition is due to their enhanced ability attribution, we performed a meditational analysis by regressing intrinsic motivation on the load manipulation and ability attribution scores. As expected, higher ability attribution predicted less intrinsic motivation (*B* = −0.47, *p*<.05), whereas the load manipulation no longer had a significant effect on intrinsic motivation after controlling for ability attribution (*B* = −0.37, *p* = .26). We also computed 95% confidence intervals of the mediation effect (load manipulation −> ability attribution −> intrinsic motivation) using the analytic distribution of a product [25]. The analysis revealed upper (−0.056) and lower (−0.903) limits that did not include 0. These results indicate that ability attribution was a statistically significant mediator of the relationship between the load manipulation and motivation (Figure 3B).

## General Discussion

Overall, the findings support our hypothesis that people automatically attribute their task performance to ability but reduce ability attribution in a controlled manner. Specifically, our experiments showed that participants attributed their task failure to ability more when cognitive resources were depleted than when they had sufficient cognitive resources. These patterns were observed even when ability is not a plausible cause of failure – participants’ awareness of other possible causal factors did not alter this increased ability attribution under cognitive load. Furthermore, our analysis indicated that the increased ability attribution due to cognitive depletion is related to impaired intrinsic motivation. Attribution literature on achievement motivation has considered attribution mainly as deliberate and effortful processes [4]. Our work, on the other hand, suggested the importance of considering the cognitive processes underlying causal attribution to achievement outcomes.

Our work has implications not only on the achievement motivation literature, but also on the literature on self-serving bias. Studies in self-serving bias have indicated that, when inferring causal reasons of one’s own behavior, people tend to attribute failure to external causes (e.g., task, teacher, mood) and success to internal causes (e.g., ability and effort; for reviews, see [26]). This is interpreted as the manifestation of self-enhancement motive [27]. However, most of the empirical studies in self-serving attributional bias collapsed different types of attributions together; they typically compute a single internal-external attribution score by subtracting external attributions (e.g., task, mood) from internal attributions (ability, effort) [26], [28]. Our work, on the other hand, highlighted the importance of examining the unique role played by ability attribution. It is also important to note that some behavioral evidence suggested that self-enhancement tendency could manifest automatically [29]–[30]. However, these studies examined self-enhancement motive in the absence of self-relevant outcome feedback. There is a large body of literature indicating that negative outcome feedback is processed automatically and has a big impact on the self [31]. In such case, self-enhancement motive may serve as a motivator of controlled process to repair the rapidly decreased self-esteem [32]–[33]. Future work would do well to examine such multifaceted function of self-enhancement motive.

A potential limitation of our research is that we used self-reports to assess attribution and intrinsic motivation. Although this is a standard way of assessing attribution and intrinsic motivation in previous literature [34]–[35], the use of self-report measures has been critiqued, due to the susceptibility of self-report data to various response biases [36]. Future work is needed to validate our findings using behavioral measurements (e.g., task-engagement to assess intrinsic motivation [37]).

Previous studies have shown that people are aware of the maladaptive consequences of ability attribution after failure [18]. Given this observation, it is a mystery why people do not stop attributing their failure to ability. Our findings provide a clue to address this issue – people do know it is maladaptive, but cannot avoid it in the first place. We hope these findings not only provide a better understanding of human causal attributional process, but also bring practical implications on how to alter maladaptive attribution in achievement settings [38].

## References

[1] Weiner B, Kukla A (1970) An attributional analysis of achievement motivation. J Pers Soc Psychol 15: 1–20.

[2] McFarland C, Ross M (1982) Impact of causal attributions on affective reactions to success and failure. J Pers Soc Psychol 43: 937–946.

[3] Perry RP, Magnusson JL (1989) Causal attributions and perceived performance: Consequences for college students’ achievement and perceived control in different instructional conditions. J Educ Psychol 81: 164–172.

[4] Weiner B (1985) An attributional theory of achievement motivation and emotion. Psychol Rev 92: 548–573. 3903815

[5] Mezulis AH, Abramson LY, Hyde JS, Hankin BL (2004) Is there a universal positivity bias in attributions? A meta-analytic review of individual, developmental, and cultural differences in the self-serving attributional bias. Psychol Bull 130: 711–747. 15367078 10.1037/0033-2909.130.5.711

[6] Sweeney PD, Anderson K, Bailey S (1986) Attributional style in depression: A meta-analytic review. J Pers Soc Psychol 50: 974–991. 3712233 10.1037//0022-3514.50.5.974

[7] Chaiken S, Trope Y (1999) Dual-process theories in social psychology. New York, NY: Guilford.

[8] Elliot AJ, Dweck CS editors. (2005) Handbook of competence and motivation. New York, NY: Guilford.

[9] Trope Y, Ben-Yair E (1982) Task construction and persistence as means for self-assessment of abilities. J Pers Soc Psychol 42: 637–645.

[10] Sedikides C, Strube MJ (1997) Self-evaluation: To thine own self be good, to thine own self be sure, to thine own self be true, and to thine own self be better. In: Zanna MP, editor. Advances in experimental social psychology (Vol. 29): New York: Academic Press. 209–269.

[11] Dweck CS, Chiu C, Hong Y (1995) Implicit theories and their role in judgments and reactions: A world from two perspectives. Psychol Inq 6: 267–285.

[12] Nicholls JG (1979) Quality and equality in intellectual development: The role of motivation in education. Am Psychol 34: 1071–1084.

[13] Plaks JE, Stecher K (2007) Unexpected improvement, decline, and stasis: A prediction confidence perspective on achievement success and failure. J Pers Soc Psychol 93: 667–684. 17892338 10.1037/0022-3514.93.4.667

[14] Cohen GL, Garcia J, Purdie-Vaughns V, Apfel N, Brzustoski P (2009) Recursive processes in self-affirmation: Intervening to close the minority achievement gap. Science 324: 400–403. 19372432 10.1126/science.1170769

[15] Dodgson PG, Wood JV (1998) Self-esteem and the cognitive accessibility of strengths and weaknesses after failure. J Pers Soc Psychol 75: 178–197. 9686458 10.1037//0022-3514.75.1.178

[16] Abramson LY, Seligman ME, Teasdale JD (1978) Learned helplessness in humans: Critique and reformulation. J Abn Psychol 87: 49–74. 649856

[17] Crocker J, Park LE (2004) The costly pursuit of self-esteem. Psychol Bull 130: 392–414. 15122925 10.1037/0033-2909.130.3.392

[18] Hareli S, Weiner B (2000) Accounts for success as determinants of perceived arrogance and modesty. Motiv Emot 24: 215–236.

[19] Gilbert DT, Silvera DH (1996) Overhelping. J Pers Soc Psychol 70: 678–690. 8636893 10.1037//0022-3514.70.4.678

[20] Frieze IH (1976) Causal attributions and information seeking to explain success and failure. J Res Pers 10: 293–305.

[21] Nasu M (1990) Causal attributions, affects and learning and behavior in an academic achievement situation. Jap J Educ Psychol 38: 17–25.

[22] Olejnik S, Algina J (2003) Generalized eta and omega squared statistics: Measures of effect size for some common research designs. Psychol. Methods 8: 434–447. 10.1037/1082-989X.8.4.434 14664681

[23] Tukey JW (1977) Exploratory data analysis. Reading, MA: Addison-Wesley.

[24] Sakurai S, Takano S (1985) A new self-report scale of intrinsic versus extrinsic motivation toward learning in children. Tsukuba Psychol Res 7: 43–54.

[25] MacKinnon DP, Fritz M, Williams J, Lockwood C (2007) Distribution of the product confidence limits for the indirect effect: Program prodclin. Behav Res Methods 39: 384–389. 17958149 10.3758/bf03193007 PMC2819369

[26] Campbell WK, Sedikides C (1999) Self-threat magnifies the self-serving bias: A meta-analytic integration. Rev Gen Psychol 3: 23–43.

[27] Sedikides C, Gregg AP (2008) Self-enhancement food for thought. Pers Psychol Sci 3: 102–116. 10.1111/j.1745-6916.2008.00068.x 26158877

[28] Duval TS, Silvia PJ (2002) Self-awareness, probability of improvement, and the self-serving bias. Pers Soc Psycholo Bull 82: 49–61. 10.1037//0022-3514.82.1.49 11811633

[29] Lench HC, Ditto PH (2008) Automatic optimism: Biased use of base rate information for positive and negative events. J Exp Soc Psychol 44: 631–639.

[30] Paulhus DL, Levitt K (1987) Desirable responding triggered by affect: Automatic egotism? J Pers Soc Psychol 52: 245–259.

[31] Baumeister RF, Bratslavsky E, Finkenauer C, Vohs KD (2001) Bad is stronger than good. Rev Gen Psychol 5: 323–370.

[32] Heimpel SA, Wood JV, Marshall MA, Brown JD (2002) Do people with low self-esteem really want to feel better? Self-esteem differences in motivation to repair negative moods. J Pers Soc Psychol 82: 128–147. 11811630 10.1037//0022-3514.82.1.128

[33] Sakaki M (2007) Mood and recall of autobiographical memory: The effect of focus of self-knowledge. J Pers 75: 421–449. 17489887 10.1111/j.1467-6494.2007.00444.x

[34] Perry RP, Stupnisky RH, Daniels LM, Haynes TL (2008) Attributional (explanatory) thinking about failure in new achievement settings. Eur J Psychol Educ 23: 459–475.

[35] Przybylski AK, Weinstein N, Murayama K, Lynch MF, Ryan RM (2012) The Ideal self at play: The appeal of video games that let you be all you can be. Psychol Sci 23: 69–76. 22173739 10.1177/0956797611418676

[36] Podsakoff PM, MacKenzie SM, Lee J, Podsakoff NP (2003) Common method variance in behavioral research: A critical review of the literature and recommended remedies. J Appl Psychol 88: 879–903. 14516251 10.1037/0021-9010.88.5.879

[37] Deci EL, Koestner R, Ryan RM (1999) A meta-analytic review of experiments examining the effects of extrinsic rewards on intrinsic motivation. Psychol Bull 125: 627–668. 10589297 10.1037/0033-2909.125.6.627

[38] Perry RP, Penner KS (1990) Enhancing academic achievement in college students through attributional retraining and instruction. J Educ Psychol 82: 262–271.

